# Modeling SARS-CoV-2 Infection in Mice Using Lentiviral hACE2 Vectors Infers Two Modes of Immune Responses to SARS-CoV-2 Infection

**DOI:** 10.3390/v14010011

**Published:** 2021-12-21

**Authors:** Chaja Katzman, Tomer Israely, Sharon Melamed, Boaz Politi, Assa Sittner, Yfat Yahalom-Ronen, Shay Weiss, Reem Abu Rass, Rachel Zamostiano, Eran Bacharach, Marcelo Ehrlich, Nir Paran, Lior Nissim

**Affiliations:** 1 Department of Biochemistry and Molecular Biology, Institute for Medical Research Israel-Canada, Faculty of Medicine, The Hebrew University of Jerusalem, Jerusalem 91120, Israel; chaja.katzman@mail.huji.ac.il; 2 Department of Infectious Diseases, Israel Institute for Biological Research, P.O. Box 19, Ness Ziona 7410001, Israel; tomeri@iibr.gov.il (T.I.); sharonm@iibr.gov.il (S.M.); boazp@iibr.gov.il (B.P.); assas@iibr.gov.il (A.S.); yfatyr@iibr.gov.il (Y.Y.-R.); shayw@iibr.gov.il (S.W.); nirp@iibr.gov.il (N.P.); 3 Faculty of Life Sciences, The Shmunis School of Biomedicine and Cancer Research, Tel Aviv University, Tel-Aviv 69978, Israel; reem.aburass@gmail.com (R.A.R.); r.zamostiano@gmail.com (R.Z.); EranBa@tauex.tau.ac.il (E.B.); marceloe@tauex.tau.ac.il (M.E.)

**Keywords:** SARS-CoV-2, COVID-19, hACE2, lentivirus, mouse model, immune response

## Abstract

Severe acute respiratory syndrome coronavirus 2 (SARS-CoV-2) caused a severe global pandemic. Mice models are essential to investigate infection pathology, antiviral drugs, and vaccine development. However, wild-type mice lack the human angiotensin-converting enzyme 2 (hACE2) that mediates SARS-CoV-2 entry into human cells and consequently are not susceptible to SARS-CoV-2 infection. hACE2 transgenic mice could provide an efficient COVID-19 model, but are not always readily available, and practically restricted to specific strains. Therefore, there is a dearth of additional mouse models for SARS-CoV-2 infection. We applied lentiviral vectors to generate hACE2 expression in interferon receptor knock-out (IFNAR1^−/−^) mice. Lenti-hACE2 transduction supported SARS-CoV-2 replication in vivo, simulating mild acute lung disease. Gene expression analysis revealed two modes of immune responses to SARS-CoV-2 infection: one in response to the exposure of mouse lungs to SARS-CoV-2 particles in the absence of productive viral replication, and the second in response to productive SARS-CoV-2 infection. Our results infer that immune response to immunogenic elements on incoming virus or in productively infected cells stimulate diverse immune effectors, even in absence of type I IFN signaling. Our findings should contribute to a better understanding of the immune response triggered by SARS-CoV-2 and to further elucidate COVID-19.

## 1. Introduction

Coronavirus disease 2019 (COVID-19), caused by severe acute respiratory syndrome coronavirus 2 (SARS-CoV-2), emerged in China in December 2019 and rapidly spread resulting in a global pandemic. Since the outbreak of the pandemic, more than 229 million cases were reported worldwide, and over 4.7 million people had succumbed to the disease as of September 2021 (WHO). Individuals infected with SARS-CoV-2 display a wide range of symptoms, ranging from very mild to critical. The most common symptoms include fever, cough, and fatigue. Severe cases include clinical manifestations such as acute respiratory distress syndrome (ARDS), cardiovascular disease, and organ failure, which are often lethal [1].

Mouse models play critical roles in studying human disease, including viral infections, and are instrumental to expedite therapeutics and vaccine development. SARS-CoV-2 entry into the host cells is mediated by the binding of the viral spike protein to its cognate human receptor angiotensin-converting enzyme 2 (hACE2) [2,3]. However, due to structural differences between the murine and human ACE2 proteins, mice are resistant to SARS-CoV-2 infection [4] with the exception of two variants of the virus, B1.351 and P.1, which were reported to infect BALB/c and C57BL/6 mice [5]. Thus, the commonly used laboratory mouse strains are not suitable for infection studies of the prevalent SARS-CoV-2 strains.

Several strategies to express hACE2 in mice and render them susceptible to SARS-CoV-2 infections were developed during the SARS-CoV outbreak in 2002 [6,7]. Similarly, it was recently demonstrated that effective SARS-CoV-2 infection is enabled in transgenic mice in which hACE2 expression is driven by the K18 [8,9,10], HFH4/FOXJ1 [11], or mouse ACE2 [12] promoters. These strains support productive viral replication in the lung that results in typical COVID-19 symptoms, such as lung pathologies and varying degrees of local inflammation.

In addition, several groups implemented adenovirus (AdV) [13,14,15] or adeno-associated virus (AAV) [16] delivery systems to generate hACE2 expression in mice lungs. This approach enabled SARS-CoV-2 replication in the lung, which resulted in immune cell infiltration and inflammation. However, transduction with AdV/AAV, which is often very immunogenic, could activate potent antiviral innate immune responses and interfere with the subsequent infection with SARS-CoV-2 [17,18]. We, therefore, implemented a lentiviral expression system to express hACE2 (Lenti-hACE2) in mice lungs since these vectors are considered moderately immunogenic [19]. As intracellular viral RNA induces an interferon (IFN) antiviral response, we used type 1 interferon receptor knock-out mice (IFNAR^−/−^) aimed to increase SARS-CoV-2 viral loads. We implemented a two sequential steps approach, in which mice were first transduced with lentiviral vectors leading to hACE2 expression, and subsequently infected with SARS-CoV-2 [19]. Our results show that mouse lung cells that were transduced with Lenti-hACE2 in vitro or in vivo became susceptible to SARS-CoV-2 infection and enabled productive viral replication. Transcriptomic analysis revealed two modes of immune response to SARS-CoV-2: (1) Exposure to SARS-CoV-2 particles even in the absence of productive infection (no replicative viruses), namely the ‘exposure model’ and (2) immune responses to productive SARS-CoV-2 infection, namely the infection model. Thus, this approach enables distinguishing the response of exposure to SARS-CoV-2 particles from the response to productive SARS-CoV-2 replication. Moreover, these Lenti-hACE2 vectors could be applied to easily generate additional SARS-CoV-2 infection models not restricted by genotype or immune background, which could enable the investigation of SARS-CoV-2 infection in vivo, for example, to study the host immune response or the effect of SARS-CoV-2 variants on infection dynamics

## 2. Materials and Methods

### 2.1. Cell Culture

HEK-293T cells (CRL-3216, ATCC) and the mouse lung epithelial type I (LET1) immortalized cell line (NR-42941, BEI Resources) were cultured in Dulbecco’s modified Eagle medium (DMEM) (01-055-1A, Biological Industries, Beit-Haemek, Israel) supplemented with 10% fetal bovine serum (FBS) (04-007-1A, Biological Industries), 1% MEM non-essential amino acids (MEM/NEAA; 01-340-1B, Biological Industries), 1% sodium pyruvate (03-042-1B, Biological Industries) 100 units/ml penicillin and 0.1 mg/ml streptomycin (03-031-1B, Biological Industries) at 37 °C with 5% CO_2_. African green monkey kidney clone E6 cells (Vero E6; CRL-1586, ATCC) and human cervical carcinoma epithelial human cells (HeLa; CRM-CCL-2, ATCC) were cultured in DMEM containing 10% FBS, MEM/NEAA, 2 mM L-glutamine, 100 units/ml penicillin, 0.1 mg/ml streptomycin, 12.5 units/ml nystatin (P/S/N), all from Biological Industries. Cells were cultured at 37 °C with 5% CO_2_ and 95% humidity.

### 2.2. Lentiviruses

The pFuGW-hACE2 (Lenti-hACE2) and pFuGW-mKate2 (Lenti-control) 3rd-generation lentiviral expression vectors were constructed using conventional Gibson assembly of either hACE2-FLAG (Sinobiological) or mKate2 inserts into the pFUGW-H1 empty vector, (Addgene Plasmid #25870) digested with BamHI and EcoRI. Plasmid sequences can be found in the GenBank database: Lenti-hACE2-FLAG (Accession Number OL870463), Lenti-control (Accession Number: OL870462).

Lenti-hACE2 and Lenti-control were propagated in HEK293T cells using the FuGENE HD transfection reagent (E2312, Promega, Madison, WI, USA). Briefly, 8 μL FuGENE HD was mixed with 100 μL OptiMEM medium (31985-047, Thermo Fisher Scientific, Waltham, MA USA) and added to a mixture of three plasmids: 0.5 μg of the pCMV-VSV-G vector (Addgene plasmid #8454), 0.5 μg of lentiviral packaging psPAX2 vector (Addgene plasmid #12260) and 1 μg of the lentiviral expression vector (pFuGW-hACE2 or pFuGW-mKate2) and incubated for 20 min at room temperature. FuGENE HD/DNA complexes were added to 1.25 × 10^6^ suspended HEK293T cells and plated in a 6-well plate. Transfection medium was replaced with fresh growth medium 18 h post-transfection. Then, 48 h post-transfection, the supernatant—containing newly produced viruses—was collected and filtered through a 0.45 μm syringe filter. For titration of infectious lentiviral stocks, HeLa cells were infected with serially diluted aliquots in the presence of 8 μg/mL polybrene in a 48-well plate and the titer was calculated based on the percent of cells expressing the mKate2 reporter gene counted under a fluorescent inverted microscope (Nikon Eclipse TE2000, Melville, NY, USA) and by staining of the fixed cells (3% paraformaldehyde (PFA), 20 min) with anti-Capsid monoclonal antibody followed by incubation with alkaline phosphatase-conjugated anti-mouse IgG (Sigma, Darmstadt, Germany) and BCIP/NBT alkaline phosphatase substrate (Sigma).

Filtered viral supernatants (10^6^ IU/ml) were used to infect 2.5 × 10^5^ LET1 cells in the presence of 8 μg/mL polybrene. The cell culture medium was replaced two days after infection. For in vivo transduction, 2.5 mL of the virus was collected and concentrated 15-fold using Amicon 100K MW cut-off ultrafiltration columns (Millipore, Burlington, MA, USA, catalog #UFC910024).

### 2.3. SARS-CoV-2

SARS-CoV-2 (GISAID accession EPI_ISL_406862) was kindly provided by the Bundeswehr Institute of Microbiology, Munich, Germany. Virus stocks were propagated (four passages) in Vero E6 cells [20]. Handling and working with the SARS-CoV-2 virus was conducted in a BSL3 facility in accordance with the biosafety guidelines of the Israel Institute for Biological Research, P.O. Box 19 Ness Ziona, Israel.

For titration of SARS-CoV-2, Vero E6 cells were seeded in 12-well plates (5 × 10^5^ cells/well) and grown overnight in a growth medium. Serial dilutions of SARS-CoV-2 were prepared in an infection medium (MEM containing 2% FBS with NEAA, glutamine, and P/S/N), and used to infect Vero E6 monolayers in triplicates (200 µL/well). Plates were incubated for 1 h at 37 °C to allow viral adsorption. Then, 2 mL/well of overlay [MEM containing 2% FBS and 0.4% tragacanth (Merck, Herzliya Pituach, Israel)] was added to each well, and plates were incubated at 37 °C, 5% CO_2_ for 72 h. The media was then aspirated, and the cells were fixed and stained with 1 mL/well of crystal violet solution (Biological Industries, Israel). The number of plaques in each well was determined, and the SARS-CoV-2 titer was calculated.

### 2.4. Western Blot Analysis and Antibodies

Western blot analyses were performed according to Melamed et al. 2004 [21] with the following modifications: nitrocellulose membranes (iBlot 2 Transfer Stacks; IB23001, Invitrogen, Carlsbad, CA, USA) were incubated with mouse monoclonal anti-ACE2 (AC18Z) (sc-73668, Santa Cruz, 1:200 dilution) overnight at 4 °C. Detection of the primary antibodies was with a secondary, horseradish peroxidase (HRP)-conjugated goat anti-mouse antibody (115-035-003, Jackson ImmunoResearch, 1:10000 dilution) for 1 h at room temperature, and with Immobilon Forte Western HRP substrate (WBLUF0500, Millipore).

### 2.5. Immunostaining

LET1 cell infection with SARS-CoV-2 was performed as previously described [20]. For immunostaining of SARS-CoV-2-infected cells, LET1 cells, Lenti-hACE2 or Lenti-control-transduced LET1 cells, or Vero E6 cells were seeded (5 × 10^4^ LET1 cells or 1.5 × 10^5^ Vero E6 cells) in an eight-well chamber (LabTek™, Nunc) and infected with SARS-CoV-2 for 24 h. Cells were then fixed with 3% PFA in PBS for 20 min, permeabilized with 0.5% Triton X-100 for 2 min, blocked with PBS containing 2% FBS, and stained with SARS-CoV-2 hyperimmune rabbit serum (in-house preparation) for 1 h. After washing with PBS, cells were incubated with Alexa Fluor 488-conjugated goat anti-rabbit at a dilution of 1:200 (A-11008, Thermo-Fisher). Nuclei were visualized by 4′,6-diamidino-2-phenylindole (DAPI). Images were acquired by an Axioskop (Zeiss) equipped with a DS-iR1 camera and NIS-elements software (Nikon). To demonstrate co-staining of hACE2 and SARS-CoV-2 in LET1 cells, the cells were similarly seeded and infected. Cells were washed with PBS, fixed with 100% methanol (−20 °C, 5 min), and permeabilized with 50% acetone/50% methanol (−20 °C, 5 min). Staining was carried out in PBS/1% BSA/0.1% Triton X-100, which was also used for washes. Antibodies employed included: anti-hACE2 (Santa-Cruz), SARS-CoV-2 hyperimmune rabbit serum (in-house preparation), Alexa Fluor-conjugated goat anti-rabbit or goat anti-mouse (fluorophores chosen according to experiment) antibodies. Images were acquired using a motorized Axiovert 200M microscope (Jena, Germany). Epifluorescence images were recorded with a CoolSnap EZ camera (Photomoterics). Images were acquired with a 100 × oil-immersion objective. Three-dimensional image stacks were acquired by the sequential acquisition of views recorded along the z-axis by varying the position of a piezoelectrically controlled stage (typical step size of 0.3 μm). SlideBook software (Version 5.0; Intelligent Imaging Innovations, Denver, CO, USA) was employed for nearest neighbors deconvolution, maximum intensity projection, and three-dimensional rendition.

### 2.6. Mouse Experiments

The animal model for SARS-CoV-2 was established by intranasal (i.n.) instillation of the lentiviruses (hACE2 or control) and three days later were similarly infected by i.n. instillation with SARS-CoV-2. Instillations were performed on anesthetized [intraperitoneal ketamine (160 mg/kg) with xylazine (6 mg/kg)] 12–14-week-old IFNAR1^−/−^ female mice (The Jackson Laboratory). SARS-CoV-2 was diluted in PBS supplemented with 2% FBS (PBF) (Biological Industries, Israel). Mouse body weight was monitored daily. All animal experiments involving SARS-CoV-2 were conducted in a BSL3 facility in accordance with the guidelines of the IIBR Institutional Animal Care and Use Committee (M-67-20). Lungs were harvested 5 dpi and stored at −80 °C for viral load determination. For RNA-seq. analyses, lungs were excised on 2 and 4 dpi and incubated in RNALater (Sigma-Aldrich) for 5 days at −70 °C before RNA preparation. For viral load determination, lungs were processed as described [22], and infectious virus quantitation was performed by plaque assay. Viral load, as well as LOD, were calculated based on volume of cell infection, dilution factor, and tissue processing volume, and presented as pfu/organ.

### 2.7. RNA-Sequencing

Raw data files can be downloaded at:

https://www.ncbi.nlm.nih.gov/geo/query/acc.cgi?acc=GSE186167 (accessed on 1 December 2021).

Mice lungs were stored at −70 °C in RNALater (Sigma Aldrich) then total RNA was isolated using an RNeasy preparation kit (Qiagen) according to the manufacturer’s protocol. RNA was stored frozen (−70 °C) until used.

RNA quality was evaluated in TapeStation, using RNA ScreenTape kit (Agilent Technologies), and quantified in Qubit apparatus (Qubit^®^ DNA HS Assay Kit, Invitrogen). Libraries for RNA sequencing were prepared using the ribosomal depletion method, due to the low RNA integrity number (RIN) that was between 6 and 7. Libraries were prepared from 10 ng RNA using SMARTer Stranded Total RNA-Seq Kit v2-Pico Input Mammalian according to the manufacturer’s recommendations. The libraries were barcoded and pooled for multiplex sequencing (1.5 pM total including 1.5% PhiX control library). The pooled DNA was loaded on a NextSeq 500 High Output v2 Kit (75 cycles) cartridge (Illumina) and sequenced on the Illumina NextSeq 500 System, using sequencing conditions: 75 cycles, single-read, and sequencing at least 35 million reads per sample.

Reads were aligned to the mouse genome, GRCm38, supplemented with the human ACE2 sequence (NM_021804.3), the mKate2 sequence (from Addgene), and the SARS-CoV-2 sequence (EPI_ISL_406862).

Differential expression analysis was performed using the DESeq2 package. Normalized counts were used for several quality control assays, such as counts distributions and principal component analysis, which were calculated and visualized in R. Those assays showed sample Lenti-hACE2_1 to be an outlier and it was thus removed from the analysis. Subsequently, since both the RNA levels of both RNA-seq data and SARS-CoV-2 genes in 2 and 4 dpi mice were similar, dpi 2 and dpi 4 samples for each model were combined (Figure S2A: $Y=1.03X-0.266,\;R^{2}=0.97$ for the exposure model, and Figure S2B: $Y=1.04X-0.282,\;R^{2}=0.97$ for the infection models).

Pair-wise comparisons were tested with default parameters, but not using the independent filtering algorithm, with two different models: (1) The exposure model (divided into two groups), and (2) The infection model (divided into three groups), as described in the Table 1 and Table 2 below.

For each pair-wise comparison, genes were defined as significant if their adjusted *p*-value (padj) was less than 0.1 (DESeq2’s default), their mean expression was above 5, and their absolute fold-change was large enough, in a mean expression-dependent manner (|log2FoldChange| > 5/sqrt(baseMean) + 0.3). The latter condition gave weight to the expression levels of the genes, so that highly expressed genes required a fold change of at least 1.2, while genes with a very low expression level would need a 5.8-fold change to pass the filtering.

All the differential expression data were subjected to gene set enrichment analysis using GSEA (https://pubmed.ncbi.nlm.nih.gov/16199517/ (accessed on 1 December 2021)) GSEA uses all differential expression data (cutoff independent) to determine whether a priori defined sets of genes show statistically significant, concordant differences between two biological states. We used the following gene set collections: Hallmark, Gene Ontology (GO), and REACTOME, all taken from the molecular signatures database MSigDB (v7.2 September 2020). In addition, we used a collection of cell type markers, extracted from the following databases: PanglaoDB (https://panglaodb.se/index.html (accessed on 1 December 2021)); CIBERSORT (https://cibersort.stanford.edu/ (accessed on 1 December 2021)); and ImmGen (https://www.immgen.org/ (accessed on 1 December 2021)).

### 2.8. Statistical Analysis

All data are presented as the means ± standard errors of the mean (SEM). Mouse weight loss comparisons between treatment groups were performed by comparison of the area under the curve (AUC) analysis. Differences in SARS-CoV-2 titers were calculated using a one-way ANOVA test corrected for multiple comparisons. All analyses were performed using GraphPad Prism software (GraphPad Software Inc., San Diego, CA, USA). *p* values of <0.05 were considered significant.

## 3. Results

### 3.1. Lenti-hACE2-Transduced Mouse Cells Support Productive SARS-CoV-2 Infection

To generate a flexible platform in which mice can be employed to study SARS-CoV-2 infection, we investigated the feasibility of a two sequential steps approach, in which mice are first transduced with Lenti-hACE2 vectors and subsequently exposed to SARS-CoV-2. To that end, we generated lentiviral expression vectors, in which hACE2 is regulated by the human ubiquitin-C promoter (hUbCp) since this promoter maintains stable and high transgene expression levels in a wide range of human and mouse cells [23].

**Figure 1:**
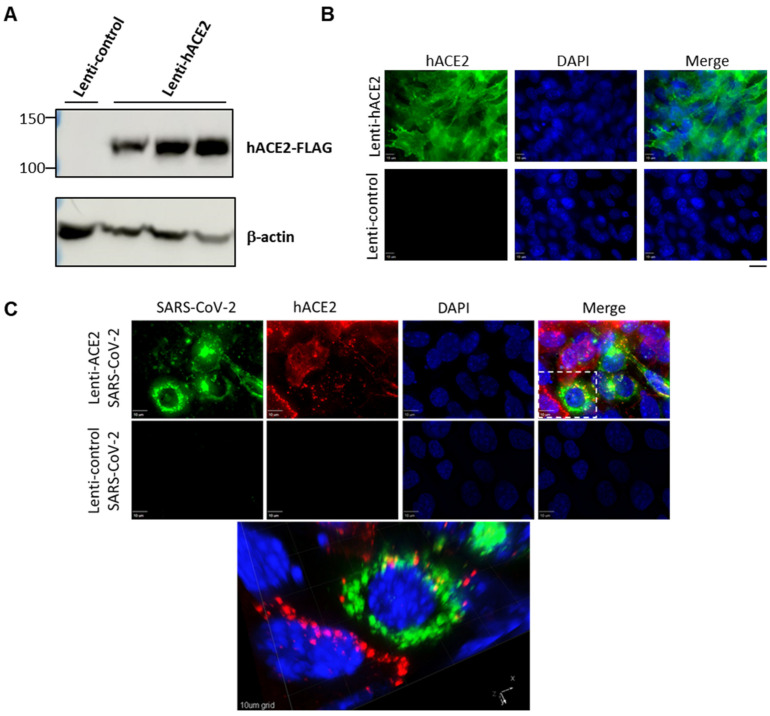
Expression of hACE2 in lentiviral-transduced murine cells allows for productive SARS-CoV-2 infection. (**A**) Western blotting analysis of lentiviral-transduced LET1 cells shows the expression of hACE2 in LET1-hACE2-transduced cells. (**B**) Immunofluorescence staining analysis of hACE2 of Lent-hACE2 and Lenti-control-transduced LET1 cells. Scale bar—20 µm. (**C**) Deconvolution microscopy of Lenti-hACE2 and Lenti-control-transduced LET1 cells infected with SARS-CoV-2. Top and lower rows depict a maximum-intensity projection of three-dimensional (3D) stacks, following deconvolution. Top row, cells were transduced with hACE-2 and infected with SARS-CoV-2; lower row, cells were transduced with control lentivirus and infected with SARS-CoV-2. Bottom micrograph is a 3D rendition of the inset appearing in the top row. 10 µm bar in upper rows, 10 µm grid in 3D rendition.

To examine whether the hACE2 lentiviral vector effectively generates hACE2 expression in murine lung cells, we transduced LET1 cells with either hACE2 expression vector (Lenti-hACE2), or a control vector in which the hUbCp drives the expression of the red fluorescent protein mKAte2 (Lenti-control). Immunoblot analysis demonstrated hACE2 expression at the expected molecular weight in LET1-hACE2 cells but not in the control cells (Figure 1A). Immunofluorescence microscopy of the LET1-hACE2 cells revealed highly efficient lentiviral transduction and abundant hACE2 expression, which demonstrated smooth peripheral staining pattern as would be expected for localization to the plasma membrane (Figure 1B). To determine whether lentiviral-induced hACE2 expression could sensitize mouse cells to SARS-CoV-2 infection, Lenti-hACE2 and Lenti-control-transduced LET1 cells were infected with SARS-CoV-2. Cells were fixed and stained with anti-SARS-CoV-2 antibodies 24 h post-SARS-CoV-2-infection. We observed co-staining of SARS-CoV-2 and hACE2 only in LET1-hACE2 cells, indicating that hACE2 expression is essential and sufficient to render LET1 cells susceptible to SARS-CoV-2 infection (Figure 1C). This was further substantiated by a significantly higher viral titer in the supernatant of Lenti-hACE2 LET1 cells compared to Lenti-control-transduced LET1 cells (Figure S1). Altogether, these results suggest that Lenti-hACE2 can support SARS-CoV-2 replication in transduced murine cells in vitro and further supports its application in vivo.

### 3.2. Lenti-hACE2-Transduced Mice Are Susceptible to SARS-CoV-2 Infection

**Figure 2:**
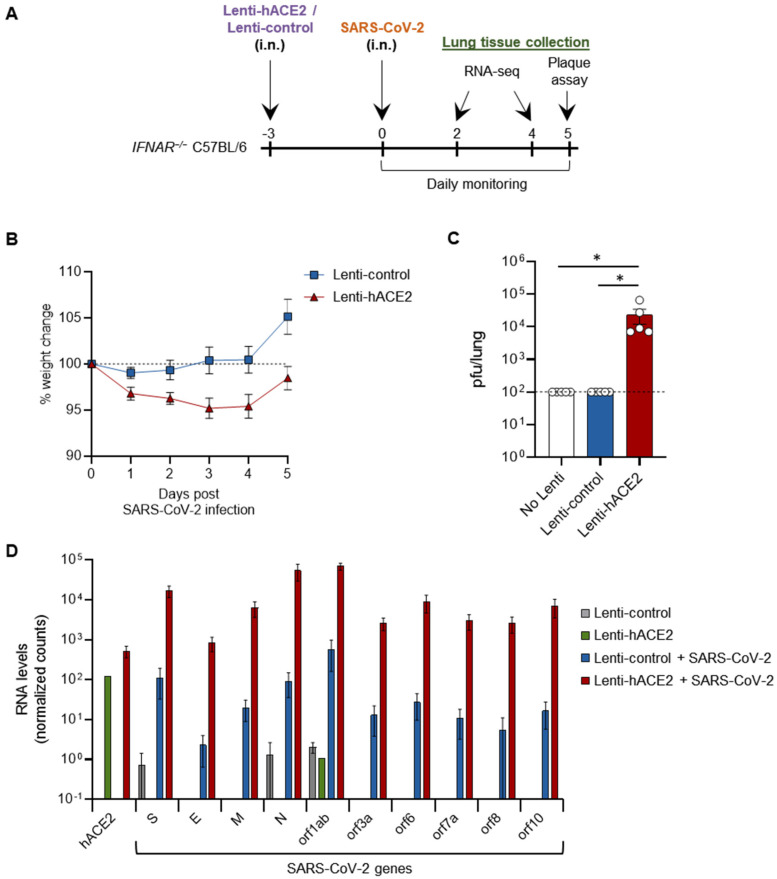
SARS-CoV-2 infection in Lenti-hACE2-transduced IFNAR^−/−^ mice. (**A**) 12–14 weeks old IFNAR^−/−^ C57BL/6 mice were intranasally transduced with hACE2 or control lentiviral vectors (Lenti-hACE2 and Lenti-control, respectively) and 3 days later infected intranasally with 2 × 10^5^ pfu SARS-CoV-2. Mice body weight was monitored daily for 5 days post SARS-CoV-2 infection (dpi). Mice were sacrificed at 5 dpi for plaque assays, and at 2 and 4 dpi for RNA-seq analysis. (**B**) Weight of Lenti-hACE2 (*n* = 5) and Lenti-control (*n* = 5) mice infected with SARS-CoV-2 was recorded for 5 days. Values are presented as the mean ± SEM *p* < 0.01 according to the area under the curve (AUC) analysis. (**C**) Virus titers in the lung were determined on Vero E6 cells. Values are presented as the mean ± SEM, *n* = 5, * *p* < 0.05 according to one-way ANOVA corrected for multiple comparisons. Dashed line represents the limit of detection. (**D**) hACE2 and SARS-CoV-2 viral genes mean expression levels ± SEM calculated from RNA-seq data of lung homogenates collected from Lenti-control (*n* = 2), Lenti-hACE2 (*n* = 1), Lenti-control infected with SARS-CoV-2 (*n* = 2; 1 at 2 dpi and 1 at 4 dpi) and Lenti-hACE2 infected with SARS-CoV-2 (*n* = 2; 1 at 2 dpi and 1 at 4 dpi) mice.

We next set out to examine whether our two sequential steps system can enable productive SARS-CoV-2 infection in vivo. For this purpose, we used type I interferon receptor knock-out (IFNAR^−/−^) mice, which are more susceptible to viral infections [24,25]. Then, 12–14 week-old IFNAR1^−/−^ mice were transduced with either Lenti-hACE2 or Lenti-control vectors (5E4 IU) by intranasal instillation and 3 days later similarly infected with 2 × 10^5^ pfu SARS-CoV-2 (Figure 2A). hACE2-transduced mice lost up to 5% of their initial weight 5 days post-SARS-CoV-2 infection (dpi), significantly more than the Lenti-control-transduced mice (*p* < 0.05, by comparing the area under the curve (AUC)) (Figure 2B). No additional signs of morbidity were observed in the hACE2-transduced/SARS-CoV-2-infected mice. Mice were sacrificed at 5 dpi and lungs were harvested. Plaque assay on lung extracts revealed infectious SARS-CoV-2 in hACE2-transduced mice, but not in Lenti-control-transduced mice (Figure 2C). In this experiment, no infectious SARS-CoV-2 particles were found in additional control of non-transduced mice infected with SARS-CoV-2 (Figure 2C). These results indicate that Lenti-hACE2 transduction of mice lungs is sufficient to support productive SARS-CoV-2 infection. Hence, this model can be utilized to further analyze SARS-CoV-2 infection outcomes in mice.

### 3.3. Immune Response to SARS-CoV-2 in Lenti-hACE2-Transduced IFNAR1^−/−^ Mice

To elucidate the cellular pathways involved in SARS-CoV-2 infection in our model, IFNAR^−/−^ mice were first transduced via intranasal instillation with Lenti-hACE2 or Lenti-control. Three days post-lentiviral transduction, mice were intranasally infected with SARS-CoV-2. Mice were sacrificed 2 and 4 dpi and lung extracts were prepared for RNA sequencing (RNA-seq). Lentivirus-transduced mice (Lenti-control or Lenti-hACE2) that were not exposed to SARS-CoV-2 were also sacrificed as a control to the SARS-CoV2 exposed mice. Data analysis showed that high levels of hACE2 mRNA were detected only in the lung homogenates of Lenti-hACE2-transduced mice (Figure 2D). Moreover, high SARS-CoV-2 viral RNA levels were detected in Lenti-hACE2-transduced mice infected with SARS-CoV-2, compared to the background, low level in Lenti-control or control mice that were not exposed to SARS-CoV-2 (Figure 2D). These observations are consistent with the fact that only Lenti-hACE2-transduced mice were productively infected with SARS-CoV-2 (Figure 2C).

**Figure 3:**
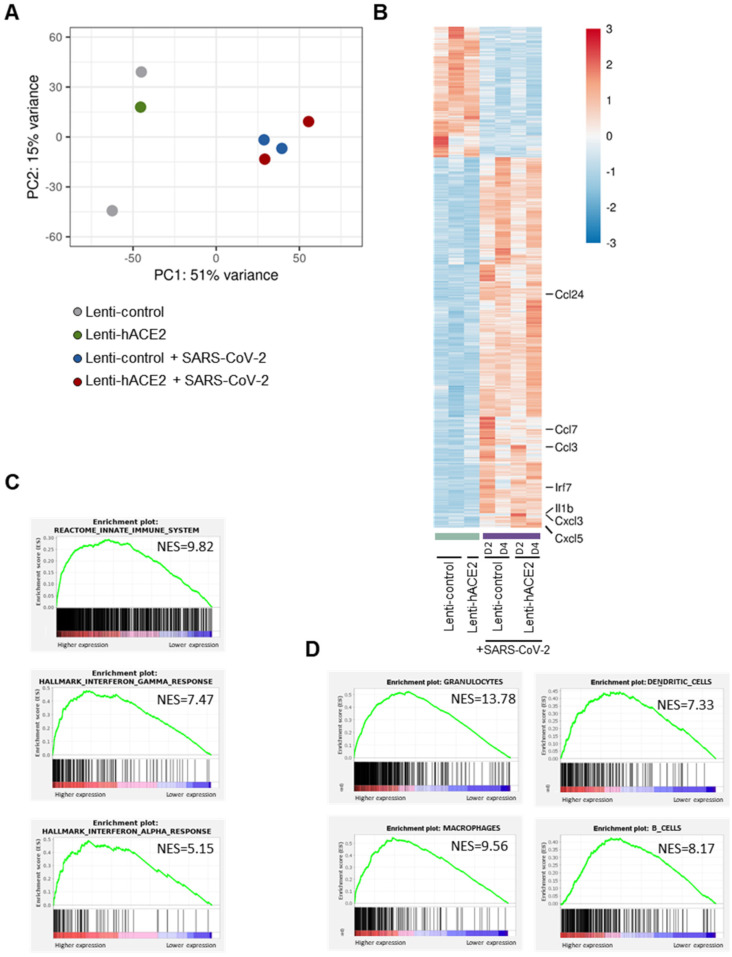
The exposure model: SARS-CoV-2 infection elicits an immune response in IFNAR^−/−^ mice. RNA-seq data of lung homogenates collected from Lenti-control (*n* = 2), Lenti-hACE2 (*n* = 1), Lenti-control infected with SARS-CoV-2 (*n* = 2; 1 at 2 dpi and 1 at 4 dpi) and Lenti-hACE2 infected with SARS-CoV-2 (*n* = 2; 1 at 2 dpi and 1 at 4 dpi) mice. (**A**) Principal component analysis (PCA) of RNA-seq data. (**B**) Heat map of significantly up- and down-regulated genes in Lenti-control and Lenti-hACE2-transduced SARS-CoV-2-infected mice versus not infected controls. (**C**) Up-regulated pathways in SARS-CoV-2-infected mice vs. not infected controls according to Reactome and Hallmark collections, FDR < 0.001. (**D**) Immune cell subtypes enriched in lungs of SARS-CoV-2-infected mice according to ImmGen classification, FDR < 0.001.

Principal component analysis (PCA) of the RNA-seq data (Figure 3A) indicated that a major contributor to differences between the groups was the exposure to SARS-CoV-2. This led us to analyze the data according to two models. For the first model, namely the exposure model, we analyzed the effect of exposure to SARS-CoV-2, irrespective of whether there was productive SARS-CoV-2 replication in the mice lungs. We, therefore, compared the SARS-CoV-2-infected animals (transduced with either Lenti-hACE2 or Lenti-control) to the non-infected group. In the second model, namely the infection model, we determined the effect of productive SARS-CoV-2 infection. For this analysis, animals were divided into three groups: (1) Lenti-control and Lenti-hACE2, the mice groups that were transduced with lentiviral vectors, but were not infected with SARS-CoV-2; (2) Lenti-control/SARS-CoV-2, the mice that were transduced with the Lenti-control vector and subsequently were infected with SARS-CoV-2; and (3) Lenti-hACE2/SARS-CoV-2, the mice that were transduced with Lenti-hACE2 were subsequently infected with SARS-CoV-2, which therefore enabled productive viral replication. To identify genes that were differentially expressed in the exposure model, we analyzed the RNA-seq data and compared gene expression patterns in lung homogenates of mice exposed to SARS-CoV-2 to mice that were not exposed to the virus. For each pair-wise comparison, genes were defined as significant if their adjusted *p*-value was less than 0.1 (DESeq2’s default), their mean expression was above 5 and their absolute fold change was correlated in a mean-expression-dependent manner. The latter condition gave weight to the expression levels of the genes so that highly expressed genes required a fold change of at least 1.2, while genes with a very low expression level would need a 5.8-fold change to pass the filtering.

Data analysis (see methods section for details) highlighted 3465 genes that were significantly upregulated and 1233 genes that were significantly downregulated in mice exposed to SARS-CoV-2 compared to non-exposed mice (Figure 3B). Interestingly, seven of the ten most highly upregulated genes were inflammatory factors, including chemokines Ccl24, Ccl7, Ccl3, Cxcl3, and Cxcl5, cytokine Il1b, and interferon regulatory factor 7 (Irf7). To identify differentially regulated pathways, we performed GSEA analysis using the Reactome and Hallmark collections.

We identified that in response to SARS-CoV-2 exposure, there was an upregulation of the innate immune system, as well as of the IFN-γ and IFN-α pathways, which are involved in antiviral responses (Figure 3C, Table S1). Following SARS-CoV-2 infection, immune cells infiltrate into the lungs of both mice [8,10,13,16] and humans [26]. To characterize the immune response in our mouse models, we identified the subtypes of immune cells that infiltrated into the lungs of both Lenti-control and Lenti-hACE2 mice that were exposed to SARS-CoV-2 (i.e., the exposure model). For this purpose, we implemented a GSEA analysis to identify gene signatures that are uniquely expressed in specific subtypes of immune cells. We found that granulocytes, macrophages, dendritic cells, and B-cells were among the most highly enriched cell types in the lungs of SARS-CoV-2-exposed mice, compared to the lungs of mice that were not exposed to SARS-CoV-2 (Figure 3D). These results indicate that the immune system is activated in response to exposure to SARS-CoV-2 particles, even in the absence of productive viral replication in the exposed mice.

After identifying the effect of exposure to SARS-CoV-2, we characterized the effects that were specifically caused by productive viral replication. For this purpose, we identified gene expression patterns that are unique to the infection model (i.e., Lenti-hACE2 mouse infected with SARS-CoV-2). The differences between the exposure and infection models were more subtle than the differences between exposed and non-exposed samples. Therefore, we performed a multi-step comparison that provides better sensitivity to differences in gene regulation compared to a direct comparison. In step 1, we found genes that are differentially expressed (padj <= 0.1) in the Lenti-hACE2/SARS-CoV-2 group (mice exposed to SARS-CoV-2 that enabled productive viral replication) compared to all mice groups that were not exposed to SARS-CoV-2 (i.e., Lenti-hACE2 and Lenti-control). This highlighted the cumulative effect on gene expression of both exposure to SARS-CoV-2 and productive SARS-CoV-2 replication. In step 2, we identified genes that were differentially expressed (padj <= 0.1) in the Lenti-control/SARS-CoV-2 group (mice exposed to SARS-CoV-2 that did not enable productive viral replication) compared to all mice groups that were not exposed to SARS-CoV-2. This step highlighted only the effect of exposure to SARS-CoV-2 on gene expression. In step 3, we identified genes that were highlighted in step 1, but not in step 2. This enabled isolating the effect of productive SARS-CoV-2 replication. Finally, we focused only on genes with expression levels that were at least 2-fold higher in the Lenti-hACE2/SARS-CoV-2 group compared to that in the Lenti-controls/SARS-CoV-2 group. This ensured that we filtered out genes with similar expression levels in the two groups, which were close to the significance threshold in the Lenti-hACE2/SARS-CoV-2 group and with padj just above 0.1 in the Lenti-controls/SARS-CoV-2 group.

**Figure 4:**
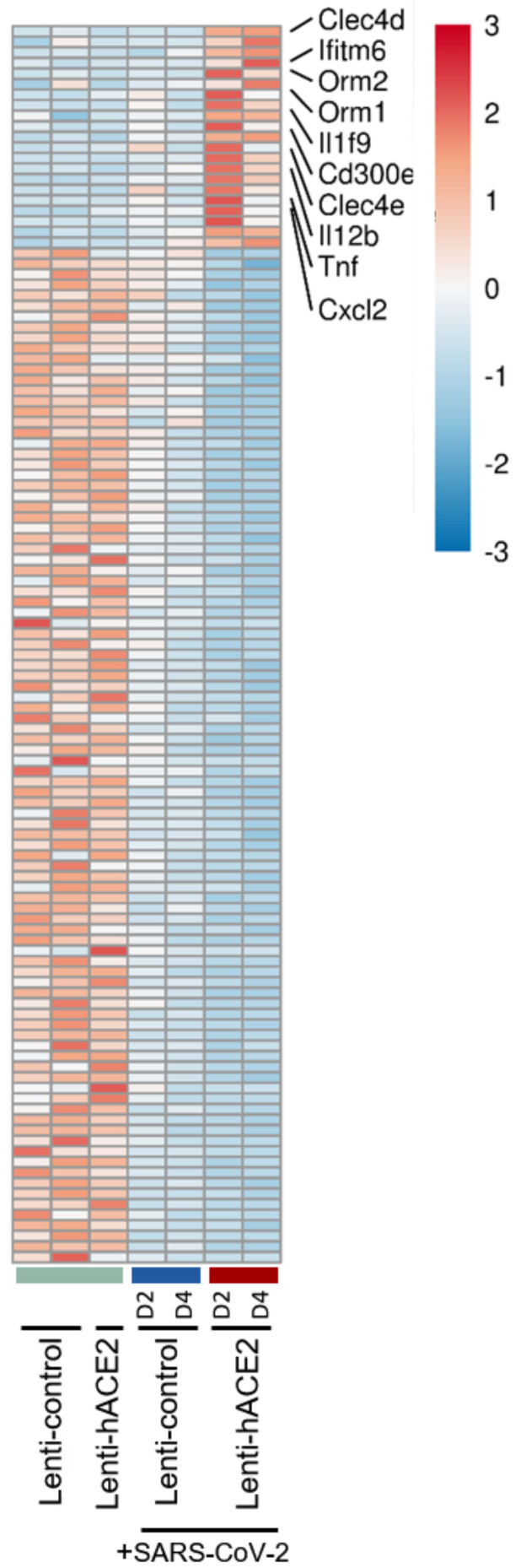
The infection model: hACE2 lentiviral transduction effect on SARS-CoV-2 infection in IFNAR^−/−^ mice. RNA-seq data of lung homogenates collected from Lenti-control (*n* = 2), Lenti-hACE2 (*n* = 1), Lenti-control infected with SARS-CoV-2 (*n* = 2; 1 at 2 dpi and 1 at 4 dpi) and Lenti-hACE2 infected with SARS-CoV-2 (*n* = 2; 1 at 2 dpi and 1 at 4 dpi) mice. Heat map of significantly up- and down-regulated genes in Lenti-hACE2-transduced SARS-CoV-2-infected mice versus control mice that were not exposed to SARS-CoV-2 (see Materials and Methods for inclusion criteria).

A total of 21 genes were upregulated and 104 downregulated specifically in the Lenti-hACE2/SARS-CoV-2 group. Among the upregulated genes (detailed in Figure 4) were the pro-inflammatory chemokine C-X-C motif chemokine ligand 2 (Cxcl2), tumor necrosis factor α TNF-α cytokine, interleukins Il12b and Il1F9, and the innate immune receptors C-type lectin domain family member (Clec) 4 E (Clec4e) and Clec4d. However, pathway analysis in this model did not yield significant results due to the small number of differentially expressed genes. Taken together, these results indicate that exposure to SARS-CoV-2 in IFNAR1^−/−^ mice results in immune response with enhanced pro-inflammatory cytokines expression, and that productive SARS-CoV-2 infection results in an additional upregulation of inflammatory factors, even in the absence of a functional type I IFN response.

## 4. Discussion

In this study, we characterized the effects of SARS-CoV-2 infection on Lenti-hACE2-transduced IFNAR1^−/−^ mice models, to achieve a more efficient SARS-CoV-2 infection and produce higher viral loads compared to wild-type mice. These mice were sensitized to SARS-CoV-2 infection by lentivirus-mediated hACE2 expression [19], as confirmed both in vitro and in vivo by detection of viral RNA, viral proteins, and infectious virion production (Figure 1, Figure 2, Figure 3 and Figure 4). RNA-seq analysis of SARS-CoV-2-infected IFNAR1^−/−^ C57BL6 mice lungs showed that lentiviral-mediated hACE2 expression does not measurably activate cellular antiviral mechanisms, which could potentially inhibit subsequent SARS-CoV-2 infection [19].

When compared to existing hACE2 transgenic mice models, lentiviral-mediated hACE2 expression enables a rapid and simple generation of mice with different genetic backgrounds. The importance of this flexibility was recently demonstrated in a study showing the influence of murine host genetic background on neurological responses to viral infection [27]. In addition, this approach can enable tissue-specific hACE2 expression, for example, by local lentivirus administration or by using synthetic gene circuits that precisely generate tissue-specific transgene expression [28,29].

Viral vectors such as AAV and adenovirus that drive hACE2 expression were previously used to generate similar mouse SARS-CoV-2 infection models [13,15,16]. However, both AAV [17] and adenovirus [18] trigger potent antiviral innate immune responses that could affect the immune response to SARS-CoV-2 infection. In contrast, PCA analysis of gene expression in the lungs of mice (Figure 3A) in our study clearly segregated samples exposed to SARS-CoV-2 from samples transduced by lentiviral vectors. These results show that exposure to SARS-CoV-2 is highly immunogenic even when the lack of hACE2 expression prevents productive SARS-CoV-2 infection and that hACE2 expression via lentiviral transduction does not trigger a significant immune response. These data support previous evidence that that exposure to SARS-CoV-2 is significantly more immunogenic than lentiviral transduction [19]. Thus, this model could be implemented as a simple methodology to generate mice models for SARS-CoV-2 infections.

Previous studies have shown that SARS-CoV-2 infection triggers the activity of key immunostimulators (for example, Cxcl3, Cxcl5, Ccl3, and Ccl7) that promote the infiltration and activation of immune cells such as granulocytes, monocytes, and macrophages [6,10,12,16,30,31,32,33]. The resulting immune response often enhances the severity of the COVID-19 symptoms [6,10,12,16,30,31,32,33]. RNA-seq analysis of lung homogenates in this study showed gene expression patterns that are concordant with these observations (Figure 3C). We found that many pro-inflammatory factors, including myeloid cell chemokines such as the aforementioned Cxcl3, Cxcl5, Ccl3, and Ccl7, were upregulated in response to SARS-CoV-2 exposure (Figure 3B). Moreover, we observed that granulocytes, macrophages, and dendritic cells infiltrated SARS-CoV-2-exposed lungs (Figure 3D). It is interesting that even though the mice are type I IFNAR knock-out, we observed differences in the immune response related to the ability of SARS-CoV-2 to infect the target cells. Thus, our data clearly highlights an IFNAR signaling-independent immune response due to SARS-CoV-2 exposure (without productive SARS-CoV-2 infection).

Recognition of pathogen-associated molecular patterns of viral origin, many of which may be generated or amplified in the context of the infected cell, is predicted to induce type I IFN responses. Indeed, type I IFN signaling and IFNAR play a role in the response to SARS-CoV-2 infection in mice models [10,12,16]. However, in AdV/AAV-hACE2-transduced mice, the loss of IFNAR activity did not significantly affect lung viral RNA levels [12,13,16]. This phenomenon may reflect the ability of SARS-CoV-2 to counter IFN-dependent antiviral responses via various molecular mechanisms [34,35,36,37], which enable its replication even in presence of IFN/IFNAR signaling. However, the loss of IFNAR activity in IFNAR^−/−^ mice is correlated with reduced induction of IFN-stimulated genes, cytokines, and chemokines, as well as attenuated inflammation at early time-points post-SARS-CoV-2 infection, compared to wild-type mice [12,16]. We, therefore, hypothesize that in this study, the lack of a functional type I IFN response contributed to the observed overall similarity of gene expression patterns in mice exposed to SARS-CoV-2 (either undergoing or lacking productive SARS-CoV-2 replication). Yet, gene expression comparison between the Lenti-hACE2-transduced mice infected with SARS-CoV-2 that enabled productive SARS-CoV-2 replication and mice groups in which there was no productive SARS-CoV-2 replication, revealed the upregulation of pro-inflammatory ligands (Cxcl2, TNF-α, Il12b, and Il1F9), as well as the innate immune receptors Clec4e and Clec4d in productively infected mice (Figure 4). These data suggest that either the increased viral load resulting from productive infection or the replication process per se, are endowed with immunostimulatory abilities, even in absence of type I IFN signaling. It should be noted that mice devoid of IFNAR-mediated signaling still express the ligands and receptors for type II (IFN-γ) and type III IFNs (IFN-λs), which may be prominently involved in the response to a viral challenge by immune or epithelial cell subsets, respectively [38,39]. The presence, functionality, and susceptibility to SARS-CoV-2-originated stimuli of the complementary IFN signaling systems in the IFNAR^−/−^ may form the molecular basis for the here-observed alterations in gene expression patterns upon exposure to SARS-CoV-2 and/or upon its replication.

The importance of understanding the roles of type I IFN in SARS-CoV-2 infection, as well as the challenge posed by the apparent variability of such roles, are exemplified by the identification of both reduced and robust type I IFN responses in COVID-19 patients [40,41,42,43], and by the variable efficacy of the use of interferon as a treatment strategy for COVID-19 [40,44]. The ability to conveniently generate models in which hACE2 is expressed in mice manipulated in expression/function of distinct immune components should contribute to the dissection of this important question.

There are limitations to this work. Mice transduced with Lenti-hACE2 developed only mild visible symptoms of the COVID-19 disease. Whether increasing the transduction efficacy and/or the load of the lentiviral vector would result in severe symptoms is yet to be determined. It will also be interesting to further investigate the effect of Lenti-hACE2 transduction in mice with a functional IFNAR signaling machinery and to evaluate the effect of antibody blockage of IFNAR signaling/type I IFN. Moreover, the observed SARS-CoV-2 viral RNA was only two orders of magnitude higher than the control mice. However, the potential of repeated administration of lentiviral vectors [45] might allow an increase in hACE2 expression in mice lungs, which could increase the local SARS-CoV-2 infection in the lungs.

In conclusion, we describe the implementation of lentiviral vectors that enable generating both in vitro and in vivo murine models for SARS-CoV-2 infection and used these models to study the effects elicited by SARS-CoV-2 in two infection scenarios: exposure and productive infection. This approach can be further exploited for a range of COVID-19 studies and drug development.

## Figures and Tables

**Table 1:** The exposure model.

Sample	Group	Lentiviral Transgene	SARS-CoV-2	SARS-CoV-2 Replication	Manuscript Annotation
Lenti-control_1	1	RFP ^1^	−	−	Lenti-control
Lenti-control _2	1	RFP ^1^	−	−	Lenti-control
Lenti-hACE2_2	1	hACE2	−	−	Lenti-hACE2
Lenti-control/SARS-CoV-2 (dpi 2)	2	RFP ^1^	+	−	Lenti-control/SARS-CoV-2
Lenti-control/SARS-CoV-2 (dpi 4)	2	RFP ^1^	+	−	Lenti-control/SARS-CoV-2
Lenti-hACE2/SARS-CoV-2 (dpi 2)	2	hACE2	+	+	Lenti-hACE2/SARS-CoV-2
Lenti-hACE2/SARS-CoV-2 (dpi 4)	2	hACE2	+	+	Lenti-hACE2/SARS-CoV-2

^1^ mKate2.

**Table 2:** The infection model.

Sample	Group	Lentiviral Transgene	SARS-CoV-2	SARS-CoV-2 Replication	Manuscript Annotation
Lenti-control_1	1	RFP ^1^	−	−	Lenti-control
Lenti-control_2	1	RFP ^1^	−	−	Lenti-control
Lenti-hACE2_2	1	hACE2	−	−	Lenti-hACE2
Lenti-control/SARS-CoV-2 (dpi 2)	2	RFP ^1^	+	−	Lenti-control/SARS-CoV-2
Lenti-control/SARS-CoV-2 (dpi 4)	2	RFP ^1^	+	−	Lenti-control/SARS-CoV-2
Lenti-hACE2/SARS-CoV-2 (dpi 2)	3	hACE2	+	+	Lenti-hACE2/SARS-CoV-2
Lenti-hACE2/SARS-CoV-2 (dpi 4)	3	hACE2	+	+	Lenti-hACE2/SARS-CoV-2

^1^ mKate2.

## Data Availability

Plasmid sequences can be found in the GenBank database: Lenti-hACE2-FLAG (Accession Number OL870463), Lenti-control (Accession Number: OL870462). The RNA-Seq data discussed in this publication have been deposited in NCBI’s Gene Expression Omnibus (Edgar et al., 2002) and upon acceptance of the manuscript will be accessible through GEO Series accession number GSE186167: https://www.ncbi.nlm.nih.gov/geo/query/acc.cgi?acc=GSE186167 (accessed on 1 December 2021).

## Supplementary Materials

The following are available online at https://www.mdpi.com/article/10.3390/v14010011/s1, Figure S1: Productive SARS-CoV-2 infection of Lenti-hACE2 transduced LET1 cells, Figure S2: Comparison between D2 and D4 mice RNA-Seq for both the exposure model (A) and the infection model (B), Table S1: Pathways upregulated in response to SARS-CoV-2 infection.
