# Supplementary material for: Modeling SARS-CoV-2 Infection in Mice Using Lentiviral hACE2 Vectors Infers Two Modes of Immune Responses to SARS-CoV-2 Infection
Source: Viruses. 2021 Dec 21;14(1):11. doi: 10.3390/v14010011 (PMC8778683; doi:10.3390/v14010011)
Supplement: Supplementary file 1 [file viruses-14-00011-s001.zip › viruses-1457485-supplementary.pdf]

**Supplementary Material**

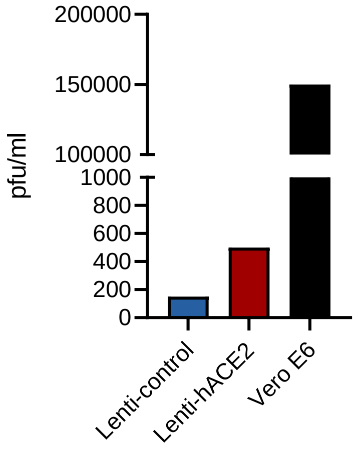


**Figure S1.** Productive SARS-CoV-2 infection of Lenti-hACE2 transduced LET1 cells**.** SARS-CoV-2 virus titers in culture media of Lenti-hACE2 and Lenti-control transduced LET1 cells or Vero E6 cells determined by plaque assay 24 hours post-infection. n=1. pfu – plaque-forming units.

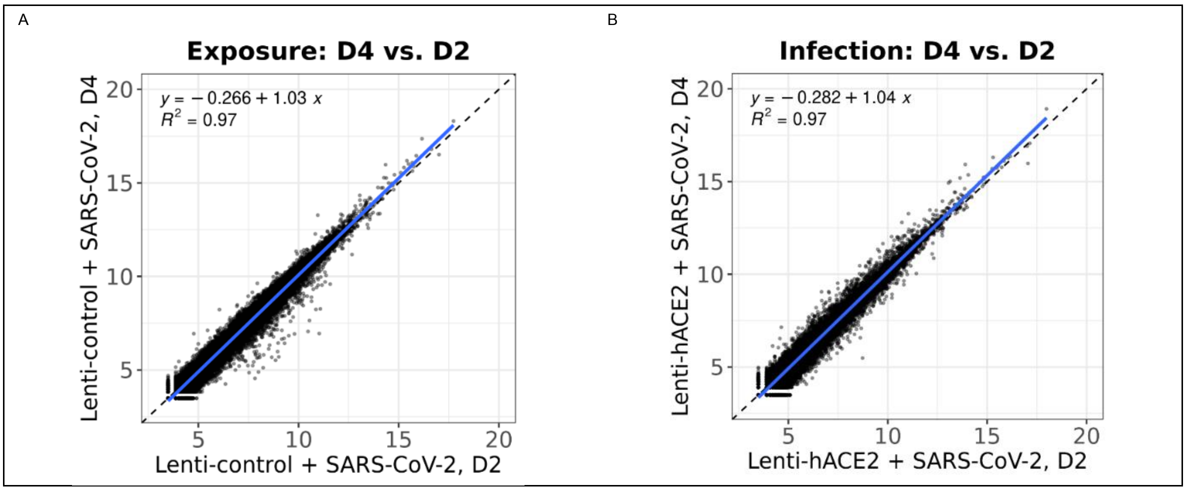


**Figure S2.** Comparison between D2 and D4 mice RNA-Seq for both the exposure model (A) and the infection model (B). Normalized counts were transformed with the vst function of the DESeq2 package and displayed as a scatter plot, comparing the different time points taken for each condition. The results of applying the 'lm' function on the data are shown as a blue line, with its equation and R squared indicated on the top left corner. The formula and R-square were added to the scatter plot image with the function 'stat_poly_eq' of the R package 'ggpmisc'

**Table S1.** Pathways upregulated in response to SARS-CoV-2 infection

| # | Reactome | NFS |
|---|---|---|
| 1 | Metabolism of RNA | 10.8 |
| 2 | Innate immune system | 9.82 |
| 3 | Cell cycle mitotic | 8.97 |
| 4 | Cell cycle | 8.78 |
| 5 | Infectious disease | 8.61 |
| 6 | Translation | 8.37 |
| 7 | Neutrophil degranulation | 8.12 |
| 8 | Cellular responses to external stimuli | 7.99 |
| 9 | HIV infection | 7.81 |
| 10 | Processing of capped intron containing pre-mRNA | 7.5 |
